# The status quo, contributors, consequences and models of digital overuse/problematic use in preschoolers: A scoping review

**DOI:** 10.3389/fpsyg.2023.1049102

**Published:** 2023-02-16

**Authors:** Chenggong Wang, Haoyue Qian, Hui Li, Dandan Wu

**Affiliations:** ^1^College of Science and Technology, Ningbo University, Ningbo, Zhejiang, China; ^2^Shanghai Institute of Early Childhood Education, Shanghai Normal University, Shanghai, China; ^3^Macquarie School of Education, Macquarie University, Sydney, NSW, Australia; ^4^Department of Early Childhood Education, Faculty of Education and Human Development, The Education University of Hong Kong, Hong Kong, Hong Kong SAR, China

**Keywords:** digital overuse, scoping review, early digital literacy, family digital environment, digital problematic use

## Abstract

Digital devices play a critical role in preschoolers’ learning and development. Despite the evidence that digital devices use may facilitate preschoolers’ learning and development, their overuse/problematic use has become a global concern as the popularity and widespread use of digital devices. This scoping review aims to synthesize the empirical evidence to identify the status quo, influential factors, developmental outcomes, and models of overuse/problematic use in preschoolers. This search has identified 36 studies published in international peer-reviewed journals during 2001–2021, converging into four common topics: the current situation, the influential factors, the consequences, and the models. First, the average percentages of overuse and problematic use across the studies collected in this research were 48.34%, and 26.83%, separately. Second, two influential factors were identified: (1) children’s characteristics and (2) parental and family factors. Third, early digital overuse/problematic use was found to have a negative impact on the following domains: (1) physical health, (2) psychosocial health, (3) problematic behaviors, and (4) cognitive development; Fourth, most relevant studies adopted general linear models, while few of them adopted experimental designs. Finally, the implications for future studies and practical improvements are also addressed.

## Introduction

Nowadays, digital technology is advancing at an unprecedented rate and dramatically shaping children’s daily lives and early development (Dong and Mertala, 2021). In particular, the COVID-19 pandemic and the associated lockdowns have forced preschoolers (ages 3–6) to learn online at home with various digital devices (Dong et al., 2020). Preschool years are a critical period of psychosocial and cognitive development and may influence life-long screen habits (Jimenez et al., 2016; Radesky and Christakis, 2016). Additionally, preschool years are also characterized by large amounts of brain plasticity. Therefore, these young minds are very sensitive and vulnerable to the effects of overusing digital devices such as smartphones, iPads, notebooks, etc. Although some studies have shown that digital devices use could promote the development of young children, such as engaging them in collaborative learning, reasoning, and problem-solving (Plowman and Stephen, 2003; Yelland, 2006). However, an increasing volume of evidence suggests that digital overuse/problematic use is evolving into a critical risk factor that threatens preschoolers’ health and well-being (Rocha and Nunes, 2020; Mallawaarachchi et al., 2022). However, there are very few empirical studies on preschoolers’ digital overuse/problematic use; thus, there is little evidence for high-stake policy-making regarding early childhood health and education matters. Therefore, a synthesis or scoping review is needed to depict the whole picture of what has been explored and reported about the topic. To meet this urgent need, this scoping review examines a wide breadth of research resources during the past two decades (2000–2021) related to the exploratory questions regarding early digital overuse/problematic use.

## Digital overuse/problematic use in preschoolers

During this digital era, children are inevitably exposed to digital media earlier in their lives and for a longer time (Dong et al., 2020). For instance, Rideout and Robb (2020) reported that 75–96% of infants use media daily, and this new generation of children is called ‘digital citizen’ or ‘digital native’ (Dong and Mertala, 2021). However, the increased screen time in preschooler’s daily life has raised concerns among public health organizations, parents, and scholars (Rocha and Nunes, 2020). Screen time refers to the time an individual spends on devices with a screen, including smartphones, tablets, computers, and televisions (Dong et al., 2020). The American Academy of Pediatrics (AAP) recommended that parents avoid digital media use in children younger than 18 to 24 months and use less than an hour per day for children aged 2 to 5 years (American Academy of Pediatrics, 2001). According to the World Health Organization (WHO), children aged below 3 are not recommended to use any digital media, and children aged 3–4 can use digital media in less than 1 h (WHO, 2019).

However, existing studies have found that preschoolers spend more screen time than the WHO standards. For example, a national survey in the United States found that toddlers (under 2 years old) spent an average of one hour of screen media per day, as reported by their parents (Lauricella et al., 2015). Coincidently, researchers also found that preschoolers consumed an average of 2–3 h per day in front of various screens (McNeill et al., 2019). All these findings have jointly confirmed that digital overuse is prevalent among preschoolers. Furthermore, Yalçin et al. (2021a) reported that problematic media use could be observed in infancy and toddler (under age 3). Domoff et al. (2020) defined ‘problematic media use’ as excessive media use that interferes with a child’s functioning, which captures dysfunctional social, behavioral, and/or academic development. It is due to excessive or maladaptive media use as evidenced by the following behaviors: (1) loss of interest in other activities; (2) preoccupation with media; (3) withdrawal from others; (4) high tolerance for media; and (5) deceptive behaviors surrounding media (Domoff et al., 2020). However, few studies have explored the factors and outcomes of digital overuse/problematic use in preschoolers. This scoping review collects all the existing studies about this topic, focusing on how early digital overuse/problematic use affects children’s development and what lessons we can learn from these studies.

In addition, family environment is an inevitable factor that is highly relevant and influential to early digital overuse/problematic use. Children’s screen time is directly associated with the related practices of their parents, such as parents’ digital addiction, parental depression, and parenting style (Lam, 2015). In particular, the status of parental digital use is also a key factor influencing children’s digital use (Dong and Mertala, 2021). Therefore, an all-around understanding of all these factors will allow us to gain a more comprehensive picture of early digital overuse/problematic use. However, most studies have focused on these factors without investigating the interactions of two or all. Therefore, another aim of this study is to summarize the factors identified in the existing studies and to propose a model for future studies.

### Theoretical framework: Developmental cascade model

**Figure 1:**
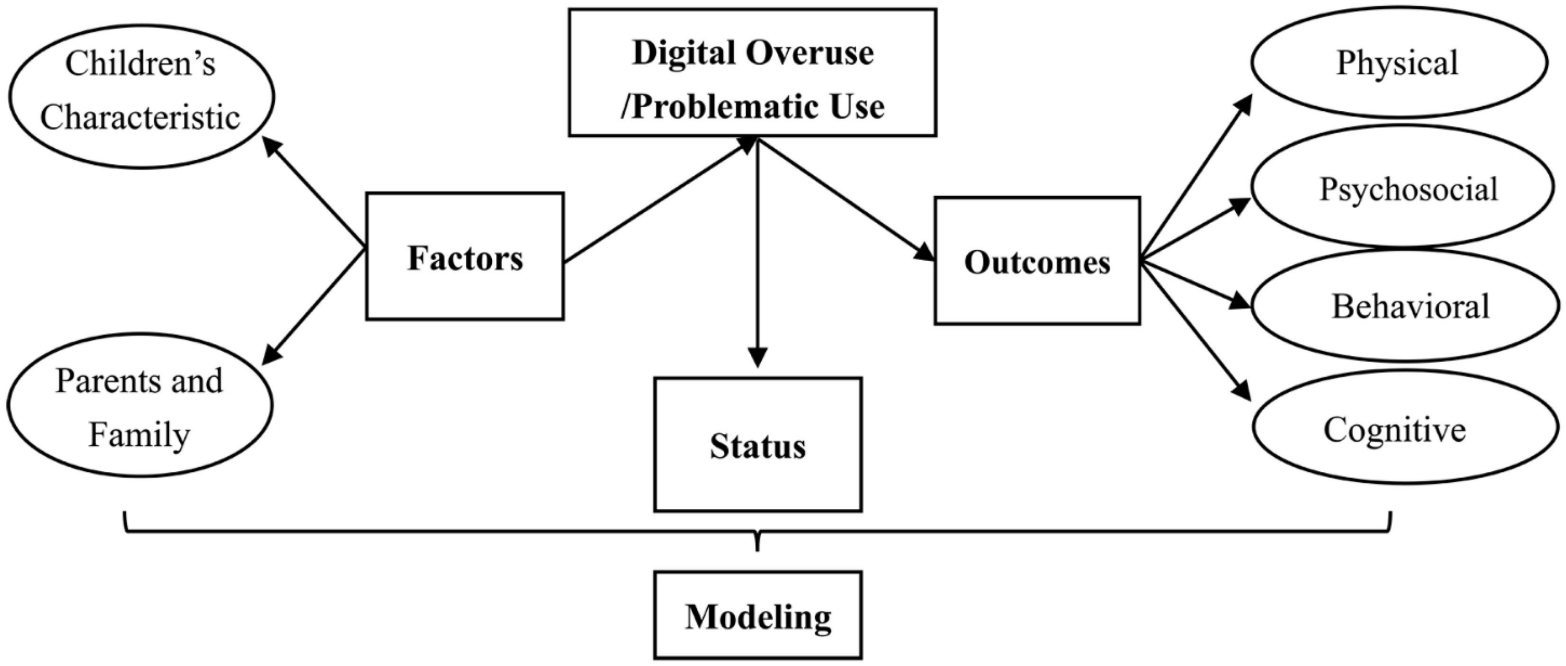
Theoretical model.

This scoping review was guided by the developmental cascade model, demonstrating how risks developed in earlier developmental periods cascade into widespread difficulties later (Gottlieb, 2007; Masten and Cicchetti, 2010). This model suggests that cumulative developmental consequences in developing systems could spread effects across levels, domains, systems, and even generations (Masten and Cicchetti, 2010). In this study, digital overuse/problematic use represents a risk from the behavioral domain. Therefore, it may lead to different developmental issues, which might, in turn, cause some negative outcomes affecting other domains (e.g., later academic achievement) through altering cognitive, neural, physical, and psychosocial development. This model is consistent with Bronfenbrenner’s bioecological framework (Bronfenbrenner, 1977, 1979), suggesting that development is a highly interactive process positioned within concentric circles of mutual influence. Based on this model (Figure 1), we have reviewed the existing literature to address the leading research problem: what are the status quo, influential factors, consequences, and models of digital overuse/problematic use in preschoolers?

## Methods

The scoping review methodology adopted in this study has been widely applied to summarize research findings for policymakers, or practitioners, identify research gaps and establish the areas for future research (Arksey and O'Malley, 2005; Levac et al., 2010). This method enables the current study to explore the width and depth of existing studies on digital overuse/problematic use (including TV, tablet, smartphone, and video game) and its factors/outcomes, identify research gaps, and establish the areas for future research. Specifically, this study has searched, identified, collected, and examined the potential sources for their relevance to the research objectives and mapped them to the key themes and concepts underpinning the research questions. In this article, we followed the 5-step framework suggested by Arksey and O'Malley (2005): (1) articulating the research questions; (2) identifying relevant studies; (3) studies selection; (4) charting the data; and (5) collating, summarizing and reporting the results.

### Phase 1: Articulating the research questions

The following questions were proposed to guide this study: (1) What is the status of digital use among preschoolers; (2) What are the influential factors of digital overuse in the preschool period? (3) What are the outcomes of digital overuse on children? (4) What statistic models have been adopted to describe the relationship among all the critical variables in this field of studies? (5) What are the research gaps in this field of study?

### Phase 2: Identifying relevant studies

The authors conceived the research questions through a series of discussions, and the first author consulted an expert in this field to identify the appropriate search terms and databases. As a result, an extensive automated search of peer-reviewed articles in the three databases (ProQuest, Web of Science, and Google Scholar) was conducted in April 2022. The literature search aimed to thoroughly identify all the research articles on “early digital use and development” published during 2001–2022. However, the studies published in 2022 did not meet the inclusion criteria. Therefore, using a full year as a cut-off point, we included studies from 2001 to 2021. Three different sets of terms with two Boolean operators (AND and OR) were utilized to search for and extract relevant literature from the databases: (screen time OR digital use OR digital overuse OR problematic digital use OR digital addiction OR TV OR smartphone OR tablet OR video game OR internet game) AND (infant OR toddler OR preschool OR prekindergarten OR kindergarten OR preschoolers OR preschoolers OR kindergarteners OR children) AND (cognition OR cognitive development OR mental health OR psychosocial health OR problematic behaviors OR behavioral problem OR physical health OR body mass index). Search terms were created *via* extensive piloting.

### Phase 3: Selecting studies

A set of criteria was employed to ensure that only full-text, English, peer-reviewed journal articles meeting the objectives of this systematic review were included. The inclusion criteria were as follows:

1. Published journal articles that focused only on preschoolers’ digital use (e.g., T.V., video game, smartphone, or tablet);
2. Results reported on digital use covering preschoolers aged 0–6 years;
3. Original studies with empirical data;
4. The sample size of the study should be more than 10;
5. English was the written language.

We excluded the articles that: (1) were not an original study, but a case report, review, commentary, erratum, or letter to the editor; (2) were original studies without empirical data, such as only semi-structured interviews or qualitative analysis; (3) studied children aged 6 years or up.

**Figure 2:**
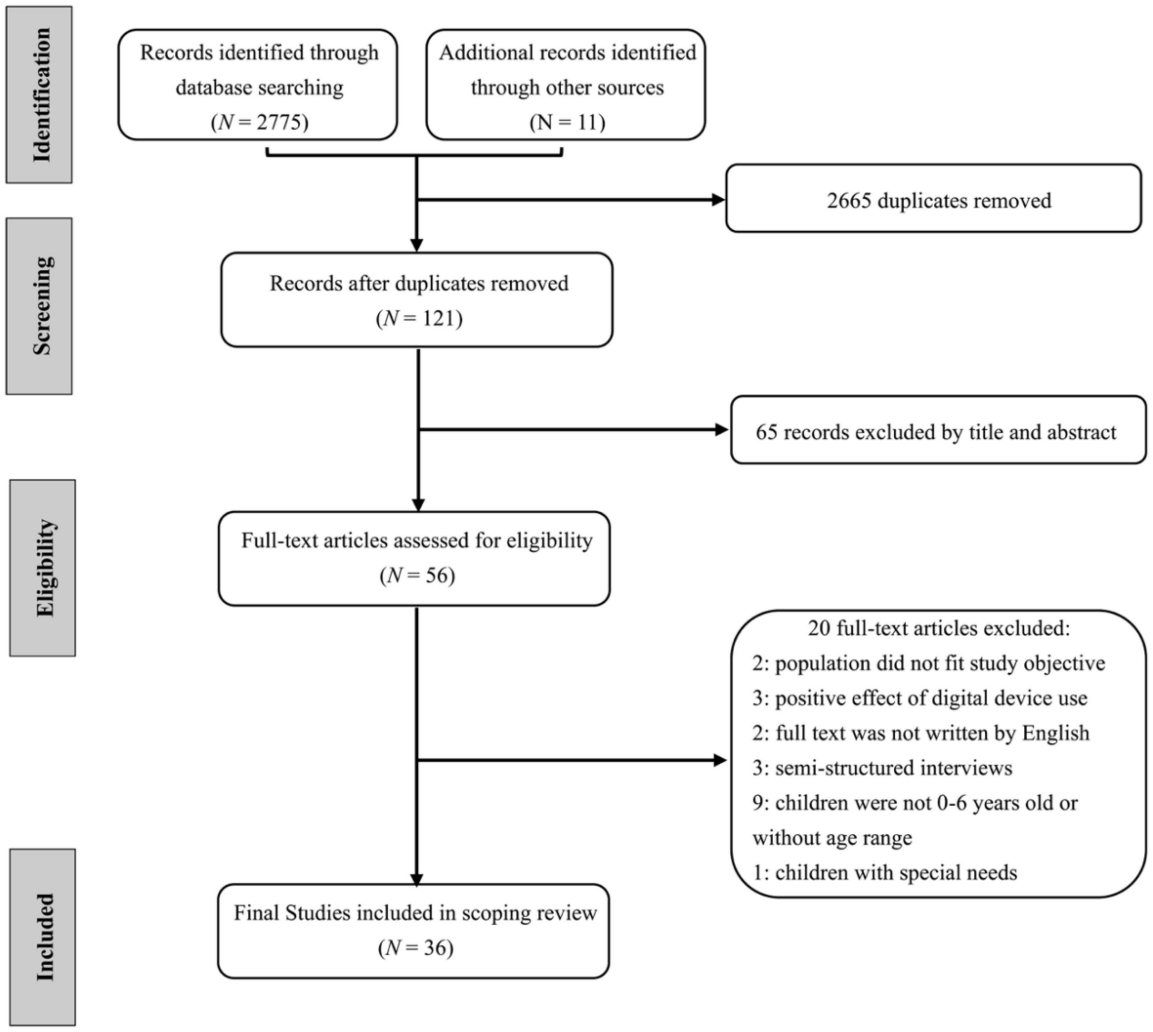
The selection of studies included in this scoping review.

As shown in Figure 2, the final search yielded 2,786 articles, of which 2,665 duplicates were removed. The first and second authors independently reviewed and selected the articles based on the inclusion criteria, and the agreement was 95.12%. Next, the authors screened full-text articles and extracted data from those that met the inclusion criteria. Due to the COVID-19 lockdowns, the authors maintained online communication throughout the full-text review process to resolve conflicts and maintain consistency. Of all the studies included for full-text review, 65 articles were excluded by title and abstract. Out of the 56 full-text studies assessed for eligibility, 20 were excluded. The authors discussed the studies that were uncertain whether to be eligible until reaching 100% agreements. Finally, a total of 36 articles were eligible for review.

### Phase 4: Charting the data

The 36 articles were charted to examine the types of research identified (see Table 1). The aggregate number of preschoolers in this scoping review was 49,126, and the sample size ranged from 38 to 20,324. The samples were recruited from 15 countries across multiple geographical regions, including Europe (*n* = 9), Asia (*n* = 14), North America (*n* = 6), and other countries/regions (*n* = 7). Most were cross-sectional studies (*n* = 31), and the others were longitudinal ones (*n* = 5). Most were general survey studies (*n* = 34), and the rest were experimental studies (*n* = 2). In particular, four articles focus on the status quo of digital use, 13 on the influential factors, and 25 on the outcomes. Among the 25 outcome-related articles, 10 focus on the influence of digital use on cognitive development, four on its influence on psychosocial development, seven on the influence on children’s behaviors, and 10 on the influence on physical health. Almost all the studies (*n* = 35) sampled preschoolers (under Age 6), whereas one study had some participants over age 6.

**Table 1:** Main characteristics of the included studies.

Author/s (Year): Country	Research topic	Sample size	Age range	Research design	Modeling	Major findings
Kim et al. (2021): South Korea	Factor	400	2–5 years	Cross-sectional	Logistic regression	Mother’s smartphone addiction positively predicts children’s early smartphone exposure. However, no correlation was found between mother’s smartphone addiction and child’s smartphone use time.
Konok et al. (2021): Hungary	Outcome	40 (study1); 56 (study2)	4–6 years	Cross-sectional	Linear regression	MTSDs use was associated with global precedence in selective attention tasks but an atypical, local precedence in a divided attention task. More importantly, playing with a digital game eliminated the advantage of selective attention over divided attention observed in the non-digital and slow digital game conditions. Besides, MTSD use was not associated with emotion recognition but with the worse theory of mind.
Madigan et al. (2020): Canada	Status	3,589	2–3 years	Longitudinal	Logistic regression	At ages two and three years, most children did not meet screen time pediatric guidelines (< 7 h per week). Besides, maternal screen time is positively associated with exceeding the screen time guidelines.
Velumani et al. (2021): India	Outcome	280	12–36 months	Cross-sectional	Linear regression	The level of screen dependency positively predicts the degree of child nourishment.
Coyne et al. (2021): USA	Factor	269	24–36 months	Cross-sectional	Structural Equation Modelling	Higher levels of media emotion regulation were associated with more problematic media use and more extreme emotions when media was removed in toddlers. Toddle’s temperament (precisely the dimensions of negative affect and surgency) influenced problematic media use and extreme emotions, and their relationship was mediated by media emotion regulation.
Lehrl et al. (2021): Germany	Factor	4,914	0–5 years	Cross-sectional	Multiple regression (including moderation effect)	Toddlers with more analogy home learning activities (e.g., parent–child activities including playing word games, reading, and counting) showed less frequent digital activities. Digital HLE activities resulted in weaker socio-emotional skills for preschoolers. Analog HLE moderated the effect of digital HLE on children’s language skills.
Xie et al. (2020): China	Factor & Outcome	1,897	3–6 years	Cross-sectional	Multiple regression	Screen time was strongly associated with preschoolers’ socioeconomic status (gender, household location, maternal education). In addition, preschoolers with screen time over 60 min per day had more behavioral problems (total and externalizing behaviors) than those less than 60 min per day.
Tay et al. (2021): Singapore	Factor	3,413	2–7 years	Cross-sectional	ANOVA	Parents’ guidance toward digital use was positively related to preschoolers’ time spent using digital media.
Anitha et al. (2021): India	Outcome	348	1.5–5 years	Cross-sectional	Chi-square test	Children under-five years of age, compared to screen time < 2 h per day, children with screen time > 2 h per day and media addiction showed more clinically developmental disorder problems and ADHD problems.
Suherman et al. (2021): Indonesia	Factor	104	3–6 years	Cross-sectional	Spearman Rho Test	Children under authoritative parenting style had less level of gadget addiction.
Cho and Lee (2017): South Korea	Factor & Outcome	303	1–6 years	Cross-sectional	Hierarchical regression (including mediation effect)	All addictive tendencies had significant positive effects on problematic behaviors and significant negative effects on emotional intelligence. Parents’ self-evaluative of their smartphone usage mediated the effect of children’s smartphone addiction proneness (such as voluntary isolation and personality distortion) on their problematic behaviors.
Özyurt et al. (2018): Turkey	Factor	76	3–6 years	Cross-sectional	Wilcoxon test	After conducting a parental training program (Triple P), parental perceived educational purposes of using digital devices changed, and the duration of their children’s digital device use decreased.
Jusienė et al. (2020): Lithuania	Outcome	190	4–5 years	Cross-sectional	Multiple linear regression	Executive functioning measures were not significantly predicted by MTSD use.
Poulain et al. (2018): Germany	Outcome	527	2–6 years	Longitudinal	Multiple regression	Baseline use of mobile phones was significantly associated with more conduct problems and hyperactivity or inattention at follow-up. Further, peer relationship problems at baseline were significantly associated with greater mobile phone use at follow-up. No significant associations were present between mobile phone use and emotional problems at baseline/ follow-up
Baek et al. (2013): South Korea	Factor	488	0–5 years	Cross-sectional	Chi-square test	Compared to mothers with high cognitive and emotional efficacy, those with low cognitive and emotional efficacy allowed their children to use smartphones more frequently.
Keefe-Cooperman (2016): USA	Factor & Outcome	492	3–5 years	Cross-sectional	Bivariate Correlation & ANOVA	Preschoolers with greater usage time of digital device had lower WPPSI-IV Visual Spatial Composite scores and Full-Scale IQ scores, on average. Lower maternal education, lower SES, and being from a historically disadvantaged background were associated with greater usage time of digital device.
Yalçin et al. (2021a): Turkey	Factor	1,245	2–5 years	Cross-sectional	Chi-square test & Logistic regression	The playing video games were partly predicted by child and family characteristics.
Yalçin et al. (2021b): Turkey	Status & Factor	1,245	2–6 years	Cross-sectional	Multiple logistic regression	The family, child, and screen use characteristics partly predicted problematic screen exposure.
Chang et al. (2018): South Korea	Status	390	2–5 years	Cross-sectional	NA	TV and smartphones were the most popular digital devices used by toddlers. Most toddlers began using smart devices at 12–24 months.
Zhao et al. (2018): China	Outcome	20,324	3–4 years	Cross-sectional	Logistic regression (including mediation effect)	Every additional hour of screen time was associated with an increased risk for poor psychosocial well-being. In addition, body mass index, sleep duration, and parent–child interaction mediated the effect of excessive screen time on children’s psychosocial well-being, among which parent–child interaction contributed the most.
Beyens and Nathanson (2019): Netherlands	Outcome	402	3–5 years	Cross-sectional	Multiple regression	Heavier television and tablet use were associated with later bedtime and later wake time, but not with fewer hours of sleep. In addition, heavier daily television use and evening smartphone use were associated with increased daytime napping. Moreover, heavier daily television use, daily and evening smartphone use, and evening tablet use were associated with poorer sleep consolidation.
Collings et al. (2018): UK	Outcome	1,338	1–3 years	Longitudinal	Linear regression	Every 1 h per day of TV viewing could predict a larger waist circumference.
Cox et al. (2012): Australian	Outcome	135	2–6 years	Cross-sectional	Hierarchical regression (including mediation effect)	Weekday TV viewing positively impacts children’s BMI *z*-score, and this effect is mediated by sedentary behavior, not the kilojoule intake during TV viewing.
Sijtsma et al. (2015): Netherlands	Outcome	759	3.4–4.4 years	Cross-sectional	Ordinary least square regression (including mediation effect)	A television in the bedroom or more televisions at home gave a higher screen time, which was associated with decreased sleep duration and resulted in higher BMI. The preschool children’s screen time and sleep duration mediated the relationship between home television ownership and BMI.
Li et al. (2021): Australia	Outcome	38	4–6.3 years	Cross-sectional	*t*-test	Compared to the ‘non-digital user’, ‘heavy-digital user’ performed poorer in the Dimensional Change Card Sort task and lower activation of the prefrontal cortex (BA 9)
Hutton et al. (2020): USA	Outcome	69	36–63 months	Cross-sectional	Spearman’s ρ	Access to child’s own smartphone and tablet was negatively correlated with Get Ready to Read score of emergent literacy and CTOPP score of processing speed. Access to child’s own smartphone and tablet was only marginally negatively correlated with the other language and literacy measures.
Cheung et al. (2017): UK	Outcome	715	6–36 months	Cross-sectional	Path analysis	Tablet use was significantly associated with a reduced overall amount of sleep and delayed sleep onset. However, tablet use was not significantly associated with frequency of night awakenings
Gülay Ogelman et al. (2018): Turkey	Outcome	162	5–6 years	Cross-sectional	Linear Regression	the use of mobile technologies was no predictive effect on the children’s social skill levels. Tablet use was not associated with social status. Smartphone use was significantly associated with lower social preferences in children.
McDaniel and Radesky (2020): USA	Factor & Outcome	183	1–5 years	Longitudinal	Structural Equation Modelling	Greater child externalizing behavior significantly predicted greater tablet use (not phone use) at follow-up *via* parenting stress (based on structural equation modeling). However, greater smartphone and tablet use did not significantly predict later externalizing behavior.
McNeill et al. (2019): Australia	Outcome	185	3–5 years	Longitudinal	Linear regression	High-dose app users at baseline had a significantly lower inhibition score at follow-up than low-dose app users; App use did not significantly predict other cognitive outcomes at follow-up
van den Heuvel et al. (2019): Canada	Outcome	893	18 months	Cross-sectional	Logistic regression	For children who used a smartphone and tablet, each additional 30-min increase in daily smartphone and tablet use was significantly associated with increased odds of parent-reported expressive speech delay. However, use was not significantly associated with other parent-reported communication delays
Lin et al. (2020): China	Outcome	161	18–36 months	Cross-sectional	Multiple regression	Smartphone and tablet use were significantly correlated with language development. However, when confounding variables were controlled for, the association was no longer significant, i.e., children who spent more time on smartphone and tablets were not more likely to have language delay.
Borajy et al. (2019): Saudi Arabia	Outcome	74	1.5–3 years	Cross-sectional	Linear regression and Logistic regression	Child’s smartphone and tablet use did not significantly influence the odds of having speech delay
Lan et al. (2020): China	Outcome	2,903	2–6 years	Cross-sectional	Linear regression	Each additional hour spent on smartphones and tablets was independently associated with a reduction in daily sleep duration of 11 and 6 min in boys and girls, respectively. Compared to non-portable devices, use of portable ones was more closely associated with short sleep duration
Moon et al. (2019): South Korea	Outcome	117	3–5 years	Cross-sectional	Spearman correlation	Smart device usage frequency positively correlated with three-year-old children’s fine motor skill development. In addition, smart device usage level was positively correlated with social development. However, smart device usage time was negatively correlated with expressive language months. No such correlations were found in children aged four to five years.
Nathanson and Beyens (2018): Netherlands	Outcome	402	3–5 years	Cross-sectional	Multiple regression	Heavier evening and daily tablet use (and, to some extent, smartphone use) were related to sleep disturbances. Besides, playing games on MEDs at bedtime was related to compromised sleep duration

MTSD, Mobile Touch Screen Devices; HLE, Home learning environment; ADHD, Attention Deficit and Hyperactivity Disorder; WPPSI-IV, Wechsler Preschool and Primary Scale of Intelligence-Fourth Edition; IQ, Intelligence Quotient; SES, Socioeconomic Status; TV, Television; BMI, Body Mass Index; BA 9, Brodmann Area 9; CTOPP, Comprehensive Test of Phonological Processing; MED, Mobile Electronic Device.

### Phase 5: Collating, summarizing, and reporting results

We extracted and collated the following essential information: author/s (year): country, research topic, sample size, age range of participator, research design, statistic model, and major findings. The first author independently reviewed the included articles and extracted data using a pre-established coding scheme. This coding scheme is used to collate and summarize the sources in four aspects, including (1) the status of children’s digital use; (2) the influential factors of digital use among preschoolers, (3) the outcomes of digital use in preschoolers, and (4) the statistic models used in this field. Any inconsistency was resolved through discussion and consensus with the co-author (s).

## Results

### The status quo of digital use

Among all the 36 studies, four have explored the status of children’s digital use, which mainly reported (1) overuse or problematic usage behaviors, (2) the frequency or children’s time spent on digital use, and (3) the types of digital devices being used. First, three studies reported severe problematic usage of digital devices. For example, Madigan et al. (2020) found that most of the 2-year-old and 3-year-old children’s screen time exceeded the line set by the WHO guidelines. Yalçin et al. (2021a) found that 22.5% of children had a problematic screen exposure score of ≥7 (defined as a high level, total score = 13), while the median score of the problematic screen exposure of the children was 4 (Interquartile Range: 3–6). Second, one study was concerned about children’s time spent on digital use. Chang et al. (2018) found that 39.3% of the 390 toddlers watched TV almost every day, while 12.0% of children used smartphones daily. In particular, more children and time had been spent on digital devices on weekends than on weekdays from a very young age (24 months old). Third, one study has focused on the digital devices used. Tay et al. (2021) found that children aged 2 to 4 spent 1.19 h per day on digital entertainment, with television and mobile phone being the most popular devices. In summary, the average percentages of overuse and problematic use across the studies collected in this research were 48.34 and 26.83%, separately (see Table 2).

**Table 2:** Digital overuse/Problematic use rates in reviewed studies.

Citation	Country	Digital overuse/Problematic use rate
Kim et al. (2021)	South Korea	NA
Konok et al. (2021)	Hungary	NA
Madigan et al. (2020)	Canada	87.9%^†^
Velumani et al. (2021)	India	82.2%^†^
Coyne et al. (2021)	USA	NA
Lehrl et al. (2021)	Germany	NA
Xie et al. (2020)	China	54.8%^†^
Tay et al. (2021)	Singapore	29.9%^†^
Anitha et al. (2021)	India	28.1%^*^
Suherman et al. (2021)	Indonesia	29.9%^*^
Cho and Lee (2017)	South Korea	12.2%^†^ (smartphone)
Özyurt et al. (2018)	Turkey	39.6%^†^ (TV)
Jusienė et al. (2020)	Lithuania	48.1%^†^
Poulain et al. (2018)	Germany	20.0%^†^ (TV)
Baek et al. (2013)	South Korea	28.6%^†^ (Smartphone)
Keefe-Cooperman (2016)	USA	NA
Yalçin et al. (2021a)	Turkey	22.5%^*^
Chang et al. (2018)	South Korea	63.1%^†^
Yalçin et al. (2021b)	Turkey	56.7%^†^
Zhao et al. (2018)	China	78.6%^†^
Beyens and Nathanson (2019)	Netherlands	NA
Collings et al. (2018)	UK	62.0%^†^
Cox et al. (2012)	Australian	NA
Sijtsma et al. (2015)	Netherlands	NA
Li et al. (2021)	Australian	NA
Hutton et al. (2020)	USA	NA
Cheung et al. (2017)	UK	NA
Gülay Ogelman et al. (2018)	Turkey	NA
McDaniel and Radesky (2020)	USA	47.1%^†^ (TV)
McNeill et al. (2019)	Australian	23.8%^†^ (App)
van den Heuvel et al. (2019)	Canada	22.4%^†^ (Mobile media devices)
Lin et al. (2020)	China	NA
Borajy et al. (2019)	Saudi Arabia	NA
Lan et al. (2020)	China	73.9%^†^
Moon et al. (2019)	South Korea	39.30%^†^
Nathanson and Beyens (2018)	Netherlands	NA
*Average*		48.34%^†^ (Overuse)
		26.83%^*^ (Problematic use)

^†^The rate of digital overuse is reported based on WHO (2019) and American Academy of Pediatrics (2001) media use guidelines of less than an hour of screen time per day. ^*^The rate of problematic use is reported based on the report of the article.

### The influential factors of digital use

Among all the 36 studies, 12 have explored the factors that influenced children’s digital use, mainly focusing on two essential aspects: (1) children’s characteristics and (2) parental and family factors (see Table 3).

**Table 3:** Findings for the factors associated with young digital overuse.

Factors	Research
Children’s characteristics	
Cultural background (Minority background)	Keefe-Cooperman (2016)
Gender (Boy)	Paulus et al. (2018); Xie et al. (2020); Yalçin et al. (2021a)
Psychological and behavioral problem	McDaniel and Radesky (2020); negative affect and surgency: Coyne et al. (2021)
Peer relationship	Poulain et al. (2018)
Number of sisters and brothers	Yalçin et al. (2021a,b)
Parental and family factors	
Parent media time	Cho and Lee (2017); Madigan et al. (2020); Coyne et al. (2021); Mother: Kim et al. (2021)
Parental attitudes toward children’s digital device use	Özyurt et al. (2018); Lehrl et al. (2021); Tay et al. (2021); Yalçin et al. (2021a)
Parenting behavior	Baek et al. (2013); Suherman et al. (2021)
Socioeconomic Status	Keefe-Cooperman (2016); Xie et al. (2020); Yalçin et al. (2021a,b)
Psychological health	Depressed: Kim et al. (2021); Parenting stress: McDaniel and Radesky (2020)

#### Children’s characteristics

Altogether eight studies found that children’s biological and sociocultural status influenced their early digital use. First, gender was found to be related to early digital use. For instance, Paulus et al. (2018) found that boys were more often and much easier to encounter computer gaming disorder than girls. Moreover, they also found that children with attention deficit hyperactivity disorder (ADHD) showed significantly higher scores in computer gaming disorder evaluation, and clinically relevant inattention scores predicted longer and more computer gaming. Later, Xie et al. (2020) revealed that boys had significantly more time on digital screens than girls. Similarly, Yalçin et al. (2021a) also reported that gender could significantly predict digital use. Second, children’s psychological and behavioral problem also affect their digital use. For example, Coyne et al. (2021) found individual’s temperament (specifically negative affect and surgency) contributed to problematic media use and extreme emotions, and their relationship was mediated by media emotion regulation. McDaniel and Radesky (2020) found that externalizing behavioral problems significantly predicted greater tablet use (not phone use) at follow-up *via* parenting stress. Parents of preschoolers with externalizing behavior are more likely to use media as a behavior modifier or babysitter (Nikken and Schols, 2015). In particular, mothers of children with externalizing behavior problems are under more pressure to raise their children and have no reasonable solutions for the externalizing behaviors their children exhibit. Therefore, allowing the child to use digital devices, such as playing games and watching animation, becomes a way to calm their children. Previous studies have verified this interpretation. For example, infants with regulatory problems (such as self-soothing difficulties and impulsive/demanding behaviors) were found to consume more TV and videos and were more likely to be given mobile devices for individual use (Radesky et al., 2016; Levine et al., 2019). Third, Paulus et al. (2018) found that peer relationship problems at baseline were significantly associated with greater mobile phone use at follow-up. This finding suggests that children with less social exposure may be prone to electronic product dependence when they grow up, and timely assessment and intervention for problematic digital use in these children is necessary. Fourth, children’s cultural backgrounds also mattered. For example, Keefe-Cooperman (2016) found that younger children with a minority background used the digital device more than European American preschoolers.

#### Parental and family factors

Seventeen studies have explored the parental factors related to early digital use, covering a wide range of factors, including parental digital use, parental attitudes toward children’s digital device use, parenting style & efficacy, family socioeconomic status (SES), and psychological health of the parents. First, parents’ digital use/problematic use was an important factor in their children’s digital overuse/problematic use. Moreover, four research studies have addressed this issue. For example, Cho and Lee (2017) found that parental smartphone usage caused smartphone problematic use proneness in their children, further leading to various problems such as interference with daily life and voluntary isolation. Madigan et al. (2020) found that maternal screen time use predicted preschoolers’ exceeding digital use guidelines. Kim et al. (2021) found that children’s first smartphone exposure was predicted by maternal smartphone addiction, while mothers’ smartphone addiction did not predict the recent smartphone use time spent by children. Recently, Coyne et al. (2021) showed that parent media time was related to the children’s problematic media use, with longer parent media time corresponding to severe problematic media use.

Second, four studies have addressed the role that parental beliefs and practices play in digital use. For instance, Özyurt et al. (2018) found that after attending a parental training program (Triple P), parents’ perceived educational purposes for digital use changed, and their children’s digital use time also declined. Later, Tay et al. (2021) found that parents’ guidance toward digital use (such as limiting screen time to 1 h per day and introducing high-quality educational programs) was positively related to the amount of time spent using digital media by preschoolers. In addition, Lehrl et al. (2021) found that even toddlers with more analogy home learning activities (e.g., parent–child activities including playing word games, reading, and counting) showed less frequent digital activities. Finally, Yalçin et al. (2021a) found that parents setting rules for preschoolers’ screen use could predict the state of children’s video game play.

Third, parenting style and efficacy also contribute to early digital use. For instance, Baek et al. (2013) found the effect of parental efficacy on preschoolers’ smartphone use. Specifically, mothers capable of solving problems and having a positive identity could control their children’s smartphone use and be aware of the positive aspects of smartphones. And the parents restricting children’s digital use helped reduce the frequency and time of smartphone use, reducing problematic levels. Recently, Suherman et al. (2021) revealed a significant relationship between parenting style and problematic digital use among preschoolers. Specifically, children under the authoritative parenting style had less digital problematic use.

Fourth, four studies jointly indicated an SES effect in early digital use. For instance, Keefe-Cooperman (2016) found that parents with lower SES were linked to the children’s greater usage time of digital devices. Next, Xie et al. (2020) reported that children’s screen time was closely correlated with their household location (urban or rural) and maternal education, as the children who lived in a rural area or with low-educated parents spent more screen time. Recently, Yalçin et al. (2021a) found that video game overuse by preschoolers could be predicted by their parental education level and the number of children in the family. Later, Yalçin et al. (2021b) confirmed that the frequency of problematic screen exposure varied in parental educational levels, maternal occupation, family type and size, and settlement type (urban or rural).

Fifth, two studies (Yalçin et al., 2021a,b) showed that the number of children in a family was related to preschoolers’ digital use. In particular, they found that if someone else was commonly playing video games at home, it resulted in a higher possibility and earlier start time point of video game playing for the children in this family. The findings suggest that the increased number of children gives caregivers less control over each child’s use of digital devices and, accordingly, high levels of digital use among preschoolers.

Finally, two studies found that parental psychological health affected preschoolers’ digital use. For example, McDaniel and Radesky (2020) confirmed an association between parenting stress and greater tablet use in preschoolers. Later, Kim et al. (2021) found that depressed mothers were more likely to have digital addiction, resulting in their children’s earlier digital use.

### The outcomes of children’s digital use

Among all the 36 studies, 26 have investigated the outcomes of children’s digital use, focusing on (1) children’s physical health, (2) psychosocial development, (3) problematic behaviors, and (4) cognitive development (see Table 4).

**Table 4:** Findings for the outcomes associated with young digital overuse.

Domain	Factors	Positive association	Negative association	No association
Physical health	Adiposity	Cox et al. (2012); Sijtsma et al. (2015); Collings et al. (2018); Velumani et al. (2021)		
	Physical activity		Cox et al. (2012); Sijtsma et al. (2015)	
	Motor skill	Moon et al. (2019)		Keefe-Cooperman (2016)
	Sleep quality	Daytime napping: Beyens and Nathanson (2019)	Sleep consolidation: Beyens and Nathanson (2019); Sleep onset: Cheung et al. (2017)	Frequency of night awakenings: Cheung et al. (2017)
	Sleep duration		Sijtsma et al. (2015); Cheung et al. (2017); Nathanson and Beyens (2018); Lan et al. (2020)	Beyens and Nathanson (2019)
Psychosocial development	Psychosocial wellbeing		Zhao et al. (2018)	
	Emotion ability		Emotional intelligence: Cho and Lee (2017)	Emotion recognition: Konok et al. (2021)
	Theory of mind		Konok et al. (2021)	
	Social skill		Gülay Ogelman et al. (2018)	
Problematic behaviors	Externalizing behavioral	Hyperactivity/ Inattention: Poulain et al. (2018); Xie et al. (2020); Anitha et al. (2021); Lehrl et al. (2021); Conduct problems: Poulain et al. (2018); Anitha et al. (2021); Lehrl et al. (2021); Aggressive behaviors: Lin et al. (2020)		McDaniel and Radesky (2020)
	Pervasive developmental disorder	Anitha et al. (2021)			Emotional problems	Poulain et al. (2018); Lin et al. (2020)		
Cognitive development	Attentional control		Konok et al. (2021)	
	Executive function		Li et al. (2021); Inhibition control: McNeill et al. (2019)	Jusienė et al. (2020); Working memory: McNeill et al. (2019)
	Visual–spatial abilities and Intelligence		Keefe-Cooperman (2016)	
	Language and literacy		Moon et al. (2019); van den Heuvel et al. (2019); Hutton et al. (2020)	Borajy et al. (2019); Lin et al. (2020)

#### Physical health

Nine articles addressed the impact of early digital use on children’s physical health, covering adiposity (Cox et al., 2012; Sijtsma et al., 2015; Collings et al., 2018; Velumani et al., 2021), physical activity (Cox et al., 2012; Sijtsma et al., 2015), motor skill (Keefe-Cooperman, 2016; Moon et al., 2019), and sleep duration and quality (Sijtsma et al., 2015; Cheung et al., 2017; Nathanson and Beyens, 2018; Beyens and Nathanson, 2019; Lan et al., 2020). In addition, four studies consistently found that early digital devices use could cause adiposity. For instance, Cox et al. (2012) reported that weekday TV viewing was positively correlated with child BMI *z*-score. More critical, sedentary behavior, not the kilojoule intake during TV viewing, mediated the positive effect of weekday TV viewing on children’s BMI *z*-score. Later, Sijtsma et al. (2015) found that longer screen time was associated with a higher BMI. They further revealed that a TV in the bedroom or more TVs at home gave a higher screen time, decreasing sleep duration and resulting in higher BMI. In addition, Collings et al. (2018) confirmed that every 1 h per day of TV viewing significantly resulted in a larger waist circumference. Recently, Velumani et al. (2021) revealed that the level of toddlers’ nourishment (such as normal weight, overweight, and obese) was positively predicted by the level of screen dependency.

Additionally, two studies revealed that early digital use would decrease children’s physical activity. Cox et al. (2012) found that weekday and weekend TV viewing was positively associated with the minutes spent in sedentary activities. Meanwhile, Sijtsma et al. (2015) also found that longer screen time was associated with less outdoor play. Another two articles explored the relationship between early digital use and motor skill development. Keefe-Cooperman (2016) revealed that screen time was not significantly correlated with the fine motor quotient. However, Moon et al. (2019) demonstrated that the use frequency of table or smartphone was positively associated with fine motor skill development in three-year-old children.

More importantly, five articles focus on the influences of digital device use on preschoolers’ sleep. Regarding sleep duration, Sijtsma et al. (2015) found that a TV in the bedroom or more TVs at home gave a higher screen time and further decreased sleep duration. Cheung et al. (2017) also found that tablet use was significantly associated with reduced sleep and delayed onset of sleep. In addition, Nathanson and Beyens (2018) found that heavier evening and daily tablet use (and, to some extent, smartphone use) contributed to sleep disturbances. Later, Beyens and Nathanson (2019) found that heavier TV and tablet use in the evening caused late bedtimes and wake-up times but did not affect sleep duration. In fact, heavier daily TV and evening smartphone use resulted in increased daytime napping. Recently, Lan et al. (2020) found that each additional hour spent on smartphones and tablets was independently associated with a reduced daily sleep duration of 11 and 6 min in boys and girls. In addition, compared to non-portable devices, the use of portable ones was more closely associated with short sleep duration.

#### Psychosocial development

Altogether four studies explored the impact of early digital use on children’s psychosocial development, which involved psychosocial wellbeing (Zhao et al., 2018), theory of mind (Konok et al., 2021), social skill development (Gülay Ogelman et al., 2018) and emotional development (Cho and Lee, 2017; Konok et al., 2021). For example, Zhao et al. (2018) found that excessive screen time led to poor psychosocial wellbeing levels. In addition, BMI, sleep duration, and parent–child interaction mediated the effect of excessive screen time on children’s psychosocial wellbeing, among which parent–child interaction contributed the most. Moreover, Konok et al. (2021) found that mobile touch screen devices (MTSD) use was associated with a worse theory of mind. In contrast, Gülay Ogelman et al. (2018) revealed that mobile technologies had no predictive effect on children’s social skill levels. Regarding emotional development, Cho and Lee (2017) found that all smartphone addictive tendencies negatively affected emotional intelligence. However, Konok et al. (2021) did not find the impact of MTSD use on preschoolers’ emotion recognition. Although most research findings suggest that preschoolers’ digital use negatively affects their psychosocial development, more studies are needed to further examine the relationship between the two.

#### Problematic behaviors

Researchers also noticed that early digital use might link with problematic behaviors, including externalizing behavior (Cho and Lee, 2017; Poulain et al., 2018; Lin et al., 2020; McDaniel and Radesky, 2020; Xie et al., 2020; Anitha et al., 2021; Lehrl et al., 2021), emotional problems (Poulain et al., 2018; Lin et al., 2020), and pervasive developmental disorder (Anitha et al., 2021). Seven of the eight studies consistently found that digital device use was associated with children’s externalizing behavior. For instance, Cho and Lee (2017) found that preschoolers’ problematic smartphone use proneness positively predicted their problematic behaviors, such as aggression, hyperactivity, and withdrawal. Laster, Poulain et al. (2018) confirmed that baseline mobile phone usage predicted more externalizing behavior at follow-ups, such as total difficulties, conduct problems, and hyperactivity or inattention. Next, Xie et al. (2020) revealed that preschoolers with screen time over 60 min per week tended to have more hyperactivity/inattention and actual difficulties. Similarly, Lin et al. (2020) found that preschoolers who spent more time on touchscreen devices were more likely to externalize aggressive behaviors. Only McDaniel and Radesky (2020) found that more digital use did not significantly predict later externalizing behavior. Recently, Anitha et al. (2021) reported that preschoolers with screen time over 2 h per day and media problematic use showed more clinically developmental disorder problems, attention deficit hyperactivity disorder related problems, and conduct problems. Similarly, Lehrl et al. (2021) found that preschoolers with a greater digital HLE experienced more difficulties and hyperactivity/inattention problems.

#### Cognitive development

Existing studies have extensively explored the impact of early digital device use on children’s cognitive development, including attention patterns (Konok et al., 2021), visual–spatial ability & intelligence (Keefe-Cooperman, 2016), executive function (McNeill et al., 2019; Jusienė et al., 2020; Li et al., 2021), and language and literacy development (Borajy et al., 2019; Moon et al., 2019; van den Heuvel et al., 2019; Hutton et al., 2020; Lin et al., 2020). For instance, Keefe-Cooperman (2016) found that preschoolers with a greater digital use time performed worse in Wechsler Preschool and Primary Scale of Intelligence-Fourth Edition (WPPSI-IV). Specifically, Konok et al. (2021) found that frequent users exhibited more global precedence in selective attention tasks but atypical, local precedence in a divided attention task. However, playing with a fast-digital game eliminated the advantage of selective attention over divided attention observed in the non-digital and slow digital game conditions.

Additionally, three articles focus on the impact of early digital use on children’s executive function. For example, McNeill et al. (2019) also revealed that high-dose app users at baseline had a significantly lower inhibition control than working memory score at follow-up than low-dose app users. However, Jusienė et al. (2020) revealed that screen use did not predict different executive abilities. Specifically, TV, computer, smartphone, and tablet use were not related to inhibitory control, working memory, and mental set shifting in preschoolers from low-risk backgrounds. Nevertheless, using functional near-infrared spectroscopy technology, Li et al. (2021) found that compared to ‘non-digital user’, ‘heavy-digital user’ performed poorer in the Dimensional Change Card Sort task, which means poorer executive function, and showed lower activation of the prefrontal cortex (BA 9). This finding indicates that neuroimaging studies might help to understand the impact of digital overuse on early executive function.

Four of the five studies showed that digital use had a negative impact on early language and literacy development. For instance, van den Heuvel et al. (2019) revealed that for children who used a smartphone and tablet, each additional 30-min increase in daily smartphone and tablet use was significantly associated with increased odds of parent-reported expressive speech delay. Additionally, Moon et al. (2019) found that digital device usage time was negatively correlated with expressive language months in three-year-old children. Later, Hutton et al. (2020) found that access to a child’s smartphone and tablet was negatively correlated with the Get Ready to Read score of emergent literacy and the Comprehensive Test of Phonological Processing score of processing speed. In addition, access to a child’s smartphone and tablet was only marginally negatively correlated with the other language and literacy measures. Similarly, Lin et al. (2020) found that digital use was significantly and negatively associated with language development. However, the association was no longer significant when confounding variables were controlled. And a ‘null result’ was reported by Borajy et al. (2019), who found child’s smartphone and tablet use did not significantly influence the odds of having speech delay. This inconsistency deserves further studies.

### Modeling the relationship between preschoolers’ digital use and development

All the studies exploring children’s digital use have focused mainly on two relationships: (1) the relationship between influential factors and early digital use; and (2) the relationship between children’s digital use and developmental outcomes. Several statistical models have been developed to model these relationships, including correlation analysis, regression analysis, moderation analysis, mediation analysis, comparison of means between groups (*t*-test/chi-square/ANOVAs), and General Linear Mixed Models (GLMM).

#### Correlation analysis

Four studies conducted correlation analysis to explore the relationship between digital use and developmental factors/outcomes. First, Keefe-Cooperman (2016) revealed a negative correlation between preschoolers’ digital use time and the WPPSI-IV and Full-Scale IQ scores. Second, Suherman et al. (2021) revealed a correlation between parenting style and problematic digital use among preschoolers. Third, Hutton et al. (2020) revealed the negative correlation between a child’s smartphone and tablet usage and emergent literacy and processing speed score. Finally, Moon et al. (2019) demonstrated the correlation between smart device usage and social development.

#### Regression analysis

Regression analysis was the most used analysis to address “digital use as a factor” and “digital use as an outcome.” Specifically, 14 studies used regression analysis to investigate the impact of digital use on children’s outcomes. For instance, Cheung et al. (2017) conducted a regression analysis on the predictive power of tablet use on sleep time and delayed sleep onset. In 2018, Gülay Ogelman et al. (2018), Nathanson and Beyens (2018), and Poulain et al. (2018), and conducted regression analyses to explore its impact. In 2019, Beyens and Nathanson (2019), Borajy et al. (2019), McNeill et al. (2019), and van den Heuvel et al. (2019) implemented general linear modeling (GLM) to model the impact of digital use. In 2020, Jusienė et al. (2020), Lan et al. (2020), Lin et al. (2020), and Xie et al. (2020) also conducted GLM to do the regression analysis. In 2021, Velumani et al. (2021) and Lehrl et al. (2021) also implemented GLM regression analysis.

Another seven studies used regression analysis to explore the influential factors of early digital use. For instance, in 2018, Paulus et al. (2018) and Poulain et al. (2018) employed GLM to analyze the influential factors. Later, Madigan et al. (2020) reported the prediction of maternal screen time use on the possibility that their children’s screen time exceeds the WHO guideline. Finally, in 2021, Kim et al. (2021) and (Yalçin et al., 2021a,b) have modeled the influences of family characteristics, child characteristics, and screen use characteristics on early digital use. In particular, Konok et al. (2021) employed the General Linear Mixed Models (GLMM) to reveal that compared to non-users, frequent MTSD user preschoolers exhibit more global precedence in the selective attention tasks.

#### Moderation/Mediation analysis

Six studies employed the mediation analysis to explore the mediator of the relationship concerning digital use. For instance, Cox et al. (2012) conducted a mediation analysis to develop a mediation model of the positive effect of weekday TV viewing on children’s BMI *z*-score. Later, Sijtsma et al. (2015), Cho and Lee (2017), and Zhao et al. (2018) employed mediation analysis to model the effects of different variables. In 2020, McDaniel and Radesky (2020) developed a structural equation model (SEM) to demonstrate the mediated prediction of child externalizing behavior on tablet use (not phone use). Recently, Coyne et al. (2021) and Lehrl et al. (2021) adopted SEM and multivariate regressions to develop mediation or moderation models.

## Discussion

This scoping review has provided important insights into the status quo, influential factors, outcomes, and statistical models reflected by the existing studies published during 2001–2021. This section will discuss the major findings and the boundaries of their application, as well as the implications for future research.

### Digital overuse/problematic use making preschoolers at risk

This scoping review identified the status quo of digital overuse/problematic use among preschoolers. First, preschoolers’ screen time has exceeded the World Health Organization guidelines and thus, they are at higher risk for addictive behavior. Second, the change in the type of digital devices varied with the spread of mobile device use and digital education (Madigan et al., 2020; Yalçin et al., 2021a). Last, the status of digital use among preschoolers is not satisfactory, as the existing studies demonstrated a relatively high rates of early digital overuse and problematic use, and the average percentage of the studies collected in this research were 48.34 and 26.83%, separately. This finding implies that our children could at risk and that swift action is needed to stop this challenging situation. In particular, the finding that boys spent more time on screens than girls and were more likely to become addicted (Xie et al., 2020) implies that boys are more vulnerable to digital overuse and need more attention, prevention, and intervention from parents and early childhood teachers.

### Parent matters

This scoping review found that parenting factors influenced preschoolers’ digital use. In particular, maternal smartphone addiction (Kim et al., 2021), parent media time (Cho and Lee, 2017; Coyne et al., 2021), parents with lower SES (Keefe-Cooperman, 2016), maternal depression (Kim et al., 2021) were positively correlated with digital overuse in preschoolers. In contrast, mother’s positive parenting style (Lehrl et al., 2021; Suherman et al., 2021; Tay et al., 2021), attitude (Özyurt et al., 2018), and self-efficacy (Baek et al., 2013) were negatively correlated with the digital overuse in preschoolers. All these findings jointly imply that parents’, especially mothers’ influences might help prevent or reduce digital overuse. Therefore, parenting programs are needed to help parents, especially mothers, understand how to cope with the challenges caused by early digital overuse/problematic use.

However, there were still some researchers holding different views toward parental factors. For example, Yalçin et al. (2021a) found that the mother’s employment status influenced the child’s digital use, whereas Kim et al. (2021) did not confirm such a relationship. In addition, Keefe-Cooperman (2016) found that children whose parents were less educated spent more time using digital devices. Moreover, Xie et al. (2020) revealed that children with lower-educated parents had more screen time. Interestingly, Kim et al. (2021) found that mothers’ digital addiction led to children’s problematic digital use instead of longer digital time. Two reasons might be associated with these mixed results. The first reason is that culture matters regarding the family structure and environment. For instance, Kim et al. (2021) conducted a study in Korea, where many mothers are full-time housewives. Thus, their employment status did not influence the family environment and their children’s digital use. However, Yalçin et al. (2021a) conducted a study in Turkey, where working mothers may influence the family environment differently. Nonetheless, future studies are needed to further explore the potential cultural influences. The second reason is relevant to the survey content, which is based on different concepts and definitions such as “digital overuse” or “problematic digital use.” Parents might have other purposes for using digital devices in preschoolers, and there might be some cultural differences. For example, Keefe-Cooperman (2016) and Xie et al. (2020) studied digital use for different purposes. For families with highly educated parents, their children who use digital devices may be more likely to meet the need for education, which benefits their development. In contrast, for parents with lower education, the purpose of using digital devices may be for leisure and entertainment or treat it as an e-babysitter, putting their children at risk of overusing digital devices. Therefore, exploration of the impact of digital device use on preschoolers’ development in the future may require distinguishing the specific purposes for which digital devices are used.

### Digital overuse/problematic use hurts early development

This scoping review has synthesized the empirical evidence to demonstrate the possible harm of digital overuse/problematic use on early childhood development. First, most studies showed that digital device overuse/problematic use might harm children’s physical health. For example, researchers found that early digital overuse could cause adiposity (Cox et al., 2012; Sijtsma et al., 2015; Velumani et al., 2021), physical inactivity (Cox et al., 2012; Sijtsma et al., 2015), poorer motor skill development (Moon et al., 2019), lack of sleep duration (Sijtsma et al., 2015; Cheung et al., 2017; Lan et al., 2020) and poor sleep quality (Sijtsma et al., 2015; Cheung et al., 2017; Nathanson and Beyens, 2018; Beyens and Nathanson, 2019; Lan et al., 2020). However, there are still some findings that remain controversial. Although most studies consistently found that digital overuse was positively associated with children’s obesity (BMI as an indicator; Cox et al., 2012; Sijtsma et al., 2015; Velumani et al., 2021), some researchers still hold different opinions. For example, Collings et al. (2018) found an insignificant correlation between time spent watching TV and BMI scores. In addition, they only found that every 1 h per day of TV viewing significantly resulted in a larger waist circumference. Given the inconsistent findings drawn from different studies, one possible explanation could be that TV, as a traditional digital device, is being used less frequently. Another possibility is that TV watching time may predict BMI by one or a combination of the following mechanisms: decreased physical activity, increased energy intake, increased sedentary behavior, and reduced sleep time. This means that there might be some mediating or moderating variables warranting further studies in the future.

However, the relationship between digital overuse/problematic use and motor skill development remains uncertain. A possible reason for this result is that in Keefe-Cooperman (2016), digital devices mainly referred to TV, while in Moon et al. (2019), digital devices changed to smartphones and tablet computers, which usually require the use of fingers for operation. We suggest that frequent use of the index finger could facilitate fine motor development in preschoolers. A previous study reported a similar finding that fine motor milestone achievements of toddlers (19–36 months) were associated with early touch screen scrolling (Bedford et al., 2016). Notably, the effects of digital overuse on children’s motor skill development still need more exploration due to insufficient empirical articles.

Second, the reviewed studies were concerned about the impact of early digital overuse/problematic use on children’s cognitive development, even though there were no agreements on how it affected each aspect of cognitive skills. For example, many studies found that early digital overuse negatively influenced children’s cognitive development, especially their language and literacy (Moon et al., 2019; van den Heuvel et al., 2019; Hutton et al., 2020). However, Lin et al. (2020) and Borajy et al. (2019) did not find the impact of digital use on children’s language and the odds of having speech delay. Another argument is surrounding the impact on executive function. For example, both Li et al. (2021) and McNeill et al. (2019) found that digital use had a negative influence on children’s executive function, whereas Jusienė et al. (2020) did not. The different tools and experimental tasks might cause these inconsistencies. Thus, further studies are also needed to settle this argument.

Third, the reviewed studies indicated that digital use for non-education purposes could cause a range of behavioral problems, including conduct problems (Poulain et al., 2018; Lehrl et al., 2021), hyperactivity/inattention (Poulain et al., 2018; Anitha et al., 2021; Lehrl et al., 2021), aggressive behaviors (Lin et al., 2020), emotional problems (Poulain et al., 2018; Lin et al., 2020), and even pervasive developmental disorder (Anitha et al., 2021). Two important factors might help prevent behavioral problems: the purpose of using digital products and the content of children’s viewing of digital products. Previous studies have demonstrated that using digital devices for educational purposes rather than entertainment would reduce behavioral problems (Fang et al., 2022). Similarly, choosing educational content rather than purely entertainment content could promote children’s imitation and learning of positive behaviors (such as prosocial behavior) rather than problematic behaviors. Therefore, using digital products for educational purposes rather than entertainment purposes might be better in reducing the negative impact of digital products on children’s behavior.

Fourth, the reviewed studies have revealed the impact of digital overuse/problematic use on children’s social–emotional development, with contradictory findings caused by using different aspects as the dependent variable. For example, Cho and Lee (2017) found that the increment in digital use reduced children’s emotional intelligence, making them hard to identify their own and others’ emotional states. In contrast, Konok et al. (2021) focused on emotional recognition and found that touchscreen use did not affect children’s performance in emotional recognition. Nevertheless, three other studies (Gülay Ogelman et al., 2018; Zhao et al., 2018; Konok et al., 2021) jointly demonstrated a great impact of early digital use on early psychosocial development and suggested that excessive digital use led to a poor level of psychosocial wellbeing, theory of mind and social skills. Therefore, we tend to conclude that the existing evidence demonstrates the negative impact of digital overuse/problematic use on early social–emotional development.

### Early digital overuse/problematic use: The models and measures

This study has reviewed the empirical studies on this topic and found various statistical models adopted in different studies, including correlation analysis, regression analysis, moderation, and mediation analysis. However, the current literature demonstrated several disadvantages regarding the statistic models. First, most of these studies adopted general linear modeling, while few adopted experimental designs. Moreover, the linear regression analysis could only determine different levels of correlation among variables, failing to confirm causality. Therefore, more empirical studies with longitudinal design should be used to explore the causal relationship among possible factors, children’s digital overuse/problematic use, and developmental outcomes in the future. Second, children’s digital overuse/problematic use is inseparable from their family, school, and living community, in which the data are inevitably nested. In this sense, a simple linear model might not be able to depict the whole picture. Therefore, the features and nature of the data should be considered in future studies. Third, the existing studies on measuring children’s digital overuse/problematic use focused on a simple aspect, such as the length of time or screen time. Future studies should consider developing a comprehensive instrument to capture the whole picture of digital use in preschoolers. Last but not least, although a certain number of studies exploring cognitive development, only one study addressed this question using a neuroimaging approach. Therefore, future studies should include behavioral and neuroscientific approaches to advance our understanding of the effects of early digital use on children’s cognitive development.

### Culture and COVID-19: Two important but overlooked factors

This scoping review indicated that two critical factors had been neglected or understudied. First, very few studies have explored the cultural influences or between-culture differences. Although early digital overuse is a global trend, the use and development of information technology in different countries are not synchronized. In addition, parents from different cultural backgrounds may hold different views on digital use and family education. For example, Eastern parents treat children’s education more strictly, while Western education is more individualized and child-centered. There is a need to explore whether culture, particularly Western versus Eastern, in itself and interaction with other factors, influences young people’s digital use. Second, the COVID-19 pandemic has thoroughly changed our lifestyle, forcing us to study or work from home. As a result, parents and their children must use digital devices for work, study, and entertainment. This has caused a sharp increase in screen time for children and their parents. However, very few studies have explored digital overuse/problematic use during the COVID-19 lockdowns and its impact on preschoolers’ longitudinal development. This research gap needs to be filled as soon as possible.

### Conclusions, limitations, and implications

As the first scoping review on digital overuse/problematic use in preschoolers, this study has synthesized the empirical evidence during the past 20 years to identify its status quo, influential factors, consequences, and models. First, the average percentage of overuse and problematic use across the studies collected in this research were 48.34 and 26.83%, separately. In particular, boys were more vulnerable to digital overuse, and parents, especially mothers, were helpful in reducing or preventing it. However, early digital overuse/problematic use for non-educational purposes may hurt preschoolers’ physical and psychosocial health and cognitive development and lead to problematic behaviors. The common methods and models adopted in this type of studies are also reviewed. In addition, this study also found a significant research gap: lacking longitudinal, neuroimaging, and multidisciplinary studies.

Nevertheless, this study has two limitations. First, it has only searched the three common full-text databases: ProQuest, Web of Science, and Google Scholar. Although very inclusive and comprehensive, the three databases might not include all the relevant studies. Other databases, such as EBSCO, JSTOR, SCOPUS, and ERIC, should also be included in the future. Second, this scoping review has focused on the English articles identified from international peer-reviewed journals. Those journals in Chinese or other languages were not included in this scoping review. Future scoping reviews should consider the language issues and include important and highly relevant journals in other important languages such as Chinese, French, and Spanish.

## Data availability statement

The original contributions presented in the study are included in the article/supplementary material, further inquiries can be directed to the corresponding author.

## Author contributions

CW and HQ contributed to data collection, processing and analysis, and original manuscript drafting. DW contributed to project conceptualization, data collection and analysis, original manuscript drafting, and supervision. HL contributed to constructive discussions and manuscript revision. All authors contributed to the article and approved the submitted version.

## Funding

This study was funded by the National Natural Science Foundation of China (Ref No. 62277037) and the Start-up Research Grant at the Education University of Hong Kong (Ref. No. RG 48/2021-2022R).

## Conflict of interest

The authors declare that the research was conducted in the absence of any commercial or financial relationships that could be construed as a potential conflict of interest.

## Publisher’s note

All claims expressed in this article are solely those of the authors and do not necessarily represent those of their affiliated organizations, or those of the publisher, the editors and the reviewers. Any product that may be evaluated in this article, or claim that may be made by its manufacturer, is not guaranteed or endorsed by the publisher.
